# Plotting of Ethylene Glycol Blood Concentrations Using Linear Regression before and during Hemodialysis in a Case of Intoxication and Pharmacokinetic Review

**DOI:** 10.1155/2015/359101

**Published:** 2015-08-06

**Authors:** Youngho Kim

**Affiliations:** Division of Nephrology, Department of Internal Medicine, University of New Mexico, 901 University Boulevard SE, Suite 150, MSC 04-2785, Albuquerque, NM 87106, USA

## Abstract

*Introduction*. As blood concentration measurement of commonly abused alcohol is readily available, the equation was proposed in previous publication to predict the change of their concentration. The change of ethylene glycol (EG) concentrations was studied in a case of intoxication to estimate required time for hemodialysis (HD) using linear regression. *Case Report*. A 55-year-old female with past medical history of seizure disorder, bipolar disorder, and chronic pain was admitted due to severe agitation. The patient was noted to have metabolic acidosis with elevated anion gap and acute kidney injury, which prompted blood concentration measurement of commonly abused alcohol. Her initial EG concentration was 26.45 mmol/L. Fomepizole therapy was initiated, soon followed by HD to enhance clearance. *Discussion*. Plotting of natural logarithm of EG concentrations over time showed that EG elimination follows first-order kinetics and predicts the change of its concentration well. Pharmacokinetic review revealed minimal elimination of EG by alcohol dehydrogenase (ADH) which could be related to genetic predisposition for ADH activity and home medications as well as presence of propylene glycol. Pharmacokinetics of EG is relatively well studied with published parameters. Consideration and application of pharmacokinetics could assist in management of EG intoxication including HD planning.

## 1. Introduction

Ethylene glycol is sweet-tasting chemical compound without odour or color found in many commercially available products such as automobile antifreeze, deicing fluids, paint, and cosmetics. American Association of Poison Control Centers reports 2070 cases of ethylene glycol exposure from automotive products alone that were treated in health care facility in 2012. Alcohols are rather benign in their original form and they become toxic by being metabolized into organic acids that consume buffering capacity with development of metabolic acidosis and cause tissue injury. Hepatic metabolism accounts for approximately 80% of ethylene glycol elimination, with the remaining 20% being eliminated unchanged in urine [1, 2]. Ethylene glycol is metabolized via alcohol dehydrogenase (ADH) to glycoaldehyde which is rapidly metabolized to glycolate, the metabolite mainly responsible for the metabolic acidosis in ethylene glycol intoxication. Glycolate is metabolized by various pathways, including one to oxalate which rapidly precipitates with calcium in various tissues and in the urine [3]. Tissue toxicity of ethylene glycol and its metabolites has been reported to show following gradient: glyoxalate > glycoaldehyde > glycolate > ethylene glycol [4]. For nephrotoxicity, glycoaldehyde and glyoxylate are the principal metabolites responsible for causing ATP depletion and phospholipid and enzyme destruction in renal tubular cell [5]. Treatment recommendation for ethylene glycol intoxication includes alcohol dehydrogenase inhibition by fomepizole (4-methylpyrazole, Antizol) to prevent biotransformation of ethylene glycol to its toxic metabolites and enhanced clearance by hemodialysis. Hemodialysis is recommended when severe metabolic acidosis (pH < 7.3) is unresponsive to therapy or renal failure exists, or if the ethylene glycol concentration is greater than 50 mg/dL unless fomepizole is being administered and the patient is asymptomatic with a normal arterial pH [1, 6]. However, ethylene glycol elimination was directly proportional to the remaining renal function as estimated by creatinine clearance, with median fractional excretion of 25.5%, and patients with normal serum creatinine concentration at the initiation of fomepizole therapy had rapid rates of renal elimination that rationalize selective hemodialysis therapy in patients treated with fomepizole when renal elimination pathway is intact [7]. As measurement of blood concentration of commonly abused alcohols is readily available in many clinical circumstances at the time of care, the equation was proposed and validated in previous publications to plan the duration of hemodialysis therapy [8, 9]. A case of intoxication was encountered during inpatient consultation service rotation that was treated with fomepizole therapy and hemodialysis. Pharmacokinetic aspect of ethylene glycol concentration during clinical course was studied and applied to predict the change of its concentration using linear regression and to estimate the required duration of hemodialysis and its efficacy.

## 2. Case Report

**Figure 1:**
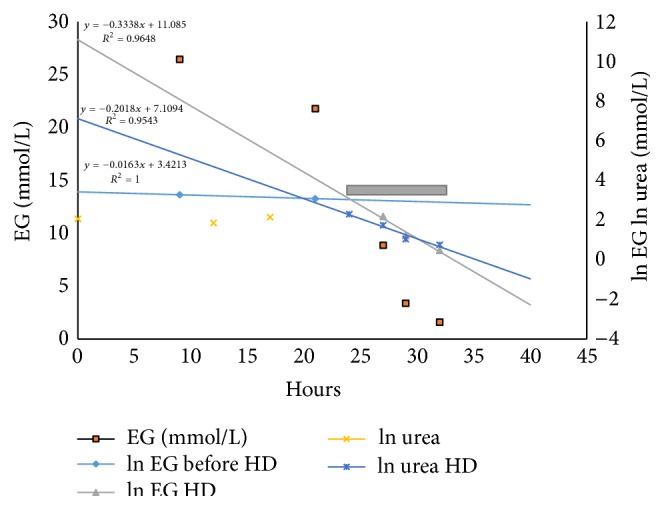
Gray bar represents hemodialysis.

A 55-year-old female with past medical history of seizure disorder, bipolar disorder, and chronic pain was admitted to ICU due to severe agitation. The patient complained of dizziness along with nausea shortly before hospitalization which was first reported to home physical therapist. There was no neurological deficit besides becoming agitated progressively over time for which she was given several doses of benzodiazepines. Her initial vital signs were blood pressure 119/75 mmHg, pulse rate 58/min, tympanic temperature 98.5, and body weight 99 kg. The second set of laboratory data after ICU admission revealed following: sodium 148 mEq/L, potassium 5.6 mEq/L, chloride 108 mEq/L, carbon dioxide 6 mEq/L, urea nitrogen 24 mg/dL, creatinine 1.85 mg/dL, calcium 8.7 mg/dL, and albumin 4.0 mg/dL. The serum anion gap was elevated at 34. Serum osmolality was not obtained. The patient was intubated for airway protection using lorazepam and rocuronium. Arterial blood gas revealed pH 7.22 and PCO_2_ 17 mmHg. Her baseline creatinine before admission was noted as 1.1 mg/dL. Blood concentrations of commonly abused alcohols were sought given anion gap metabolic acidosis and additional history of psychosocial issues from family. Urinalysis was negative for crystals. Ethylene glycol level became available 169 mg/dL (26.45 mmol/L) 19 hours after admission and other alcohols were negative. Glycolic acid or glyoxylic acid blood concentration was not obtained. Quantification of consumed ethylene glycol was not possible due to the lack of reliable consumption history. Plotting of blood concentrations of ethylene glycol and urea and their corresponding natural logarithm with trend lines using linear regression function is shown in Figure 1. Fomepizole therapy was initiated and, within 2 hours, hemodialysis followed. The patient was treated using Polyflux Revaclear MAX dialyzer (Gambro, 1.8 m^2^ membrane surface area) via right internal jugular vascular catheter. Blood flow and dialysate flow were set 300–400 mL/min and 1.5 times blood flow, respectively. Total volume treated was 138.6 L for 8 hours with average blood flow 290 mL/min. The patient was maintained on continuous IV drip of lorazepam for sedation along with several doses of IV phenytoin for subtherapeutic drug level noted upon admission.

## 3. Discussion

During hemodialysis, solute elimination occurs via the first-order kinetic process, and the distribution of a drug in a dialyzed, renal failure patient can be expressed by the one-compartment model [7, 10]. Change of concentration over time in first-order kinetics could be expressed as below and integrated to encompass the times of sampling and measurement to evaluate kinetic process and natural logarithm of concentration change would show linear relation over time:

(1) rate−dCdt=kCdCC=−k·dt∫dCC=∫−k·dt∫0t1C·dC=−k∫0tdtln⁡Ct−ln⁡Co=−ktln⁡Ct=−kt+ln⁡Co,

where *C* is concentration, *t* is time, and *k* is elimination rate constant.

Ethylene glycol concentrations during hemodialysis show exponential decrease over time and their corresponding natural logarithm exhibits linear relation suggesting first-order kinetic elimination of ethylene glycol. Fomepizole therapy was started only 2 hours prior to hemodialysis which makes its impact on ethylene glycol concentration in our case minimal. Total elimination rate constant before hemodialysis, sum of renal and hepatic elimination by ADH (*k* ^total  before  HD^ = *k* ^renal^ + *k* ^ADH^), is calculated to be 0.0163 h^−1^, expressed as a slope of function of natural logarithm of ethylene glycol concentrations over time. Ethylene glycol elimination is known to follow first-order kinetics in the absence of treatment, with an estimated serum half-life of between 3 and 9 hours [7, 11] and it was 42.5 hours in our case. *k* ^renal^ was estimated as 0.0128 h^−1^ based on creatinine clearance by Cockcroft-Gault formula, suggested fractional excretion, and volume of distribution. It would leave much smaller *k* ^ADH^ than previously observed which could be due to her genetic predisposition for ADH activity and home medications including morphine and methylphenidate that were known to inhibit ADH activity in vitro [12, 13]. Interestingly, propylene glycol level became positive later in the course and its concentration peaked to 88 mg/dL. Continuous IV drips of lorazepam and phenytoin were thought to be sources. Propylene glycol, though considered generally safe, can cause intoxication when large quantities are ingested. Several cases of lactic acidosis after inadvertent propylene glycol intoxication were reported in patients with renal dysfunction [14]. Propylene glycol shares the same metabolic pathway with ethylene glycol and may compete for ADH interfering with hepatic elimination of ethylene glycol in our case. Total elimination rate constant during hemodialysis was significantly increased to 0.3338 h^−1^. Manufacturer's box inlet indicates in vitro urea clearance of 293 mL/min with blood flow at 300 mL/min which we achieved close in our case. Actual urea elimination rate constant and clearance in our case were 0.2018 h^−1^ and 133.5 mL/min, respectively, based on Watson estimate of total body water and the difference of urea clearance could be partly from in vivo urea generation in catabolic state of critically ill patient. Using the assumption that toxic alcohols would have a dialysis clearance similar to urea and the volume of distribution of toxin is the total body water as determined by the Watson formula, the following equation was proposed to estimate the required dialysis time in hours to reach a 5 mmol/L toxin concentration target [8]:

From the above equations, ln⁡[*C*]*t* = −*kt* + ln⁡[*C*]*o* and since *K*(clearance) = *k* · *Vd*,

(2) $$\begin{array}{c} -\frac{V\ln\left(5/Co\right)}{0.06K}, \end{array}$$

where *V* is Watson estimate of total body water, *Co* is the initial concentration (mmol/L), *K* is clearance of toxin that is assumed to be 80% of the manufacturer-specified dialyzer urea clearance (mL/min) at the initial observed blood flow rate to allow estimates to be made at the start of dialysis [8], and 0.06 is conversion factor to have product in hour. There was significant difference in the manufacturer's in vitro urea clearance, actual urea, and ethylene clearance during hemodialysis, the equation produced 4.3 hours of required dialysis time which was overestimated approximately by 1 in our case when compared to linear regression plot of actual concentration change of ethylene glycol. Half of the hemodialysis after initial 4 hours out of total 8-hour treatment did not contribute much in regard to ethylene glycol concentration reduction clinically.

In conclusion, plotting of ethylene glycol blood concentrations and their natural logarithm showed that ethylene glycol elimination during hemodialysis followed first-order kinetics and predicted the change of its concentration well. In our case, hepatic elimination of ethylene glycol by ADH was minimal which could be related to genetic predisposition for ADH activity and home medications as well as presence of propylene glycol. Pharmacokinetics of ethylene glycol is relatively well studied as one of the commonly abused alcohols with published parameters. Consideration and application of pharmacokinetics could assist with hemodialysis planning in clinical practice.
